# Effects of Porcine Zona Pellucida Immunocontraception on Mare Body Condition and Foaling Season Length in Two Western Wild Horse Populations

**DOI:** 10.3390/ani14233550

**Published:** 2024-12-09

**Authors:** Allen T. Rutberg, Kayla A. Grams

**Affiliations:** 1 Cummings School of Veterinary Medicine at Tufts University, 200 Westboro Road, North Grafton, MA 01536, USA; 2 Science and Conservation Center, 2100 South Shiloh Road, Billings, MT 59106, USA

**Keywords:** *Equus caballus*, wildlife contraception, wild horse, feral horse, body condition scoring, foaling season

## Abstract

Wildlife managers and the public have expressed considerable interest in the use of contraception to help manage the populations of wild horses and burros (*Equus caballus* and *E. asinus*). Field testing has shown that two preparations of the porcine zona pellucida (PZP) vaccine, a simple emulsion (ZonaStat-H) and PZP-22 (which supplements ZonaStat-H with a controlled-release component) effectively prevent pregnancy in individual mares and can substantially reduce population foaling rates. To determine whether some PZP preparations might have secondary effects that harm treated mares or their foals, we examined the effects of PZP-22 vaccinations and the follow-up boosters of either PZP-22 or ZonaStat-H on adult female body condition, foaling season, and foal mortality in two wild horse herds in the western USA, Cedar Mountains Herd Management Area, Utah (CM; 2008–2015), and Sand Wash Basin Herd Management Area, Colorado (SWB; 2008–2014). At both sites in every study year, summer body condition scores improved faster in mares without foals than mares with foals (*p* < 0.001; CM, *n* = 234; SWB, *n* = 172), but PZP treatments did not affect mare body condition apart from their contraceptive effects. Births to mares treated with PZP within the previous three years were delayed and spread out over the foaling season, but foal mortality rates through the first and second year were low, unrelated to date of birth, and virtually identical for the foals of PZP-treated and untreated mothers (all comparisons n.s.; CM, *n* = 775, SWB, *n* = 640). Thus, in these two populations, we found no evidence that changes in reproductive timing associated with PZP treatments were harmful to either mares or foals.

## Simple Summary

Herds of free-roaming, unowned horses can grow rapidly on rangelands with limited resources. Contraception can offer a humane, publicly acceptable method for slowing or stopping horse population growth and protecting the range. The best-tested and most widely used contraceptive for wild horses, porcine zona pellucida (PZP), a protein-based vaccine, has successfully stabilized or reduced free-roaming horse herds in different environments. While extensive research has shown that the PZP vaccine is safe, concerns still exist about its possible effects on the behavior and well-being of treated mares, and especially the survival of their offspring. Reporting the results of an eight-year study of PZP contraception in wild horses at two sites in the western USA, we show here that mares without foals improved the body condition faster than mares with foals. We also found that the foaling seasons of the PZP-treated herds were later in the year and more spread out. However, deaths of the foals born to PZP-treated mares through their second year of life were very rare and no more frequent than those of foals born to untreated mares. Thus, at our study sites, the timing of breeding was changed by PZP treatments, but we found no evidence that these changes were harmful either to the treated mares or their foals.

## 1. Introduction

The use of immunocontraceptive vaccines to limit population growth in captive and free-roaming wildlife is spreading [1,2]. These vaccines, predominantly GonaCon^TM^ and porcine zona pellucida (PZP), have been tested on and applied to free-roaming horses (*Equus caballus)* [3,4,5,6,7], white-tailed deer (*Odocoileus virginianus*) [8,9], African elephants (*Loxodonta africana*) [10], prairie dogs (*Cynomys ludovicianus*) [11], and over 100 species (mostly ungulates) held captive in zoos [1,12]. A substantial number of these studies report data on vaccine effectiveness in the field at the individual level. Several also examine effects of immunocontraceptive administration on population dynamics [13,14,15].

Data on health and behavioral effects of immunocontraception are scarcer, and are sometimes not distinguished from the consequences of not having offspring. Among the phenomena observed in association with the application of immunocontraceptives are changes in body condition among immunocontraceptive-treated females, lengthening of mating and birth seasons, increased mating behavior directed at contraceptive-treated females, and potential disruptions of social structure and time budgets [16,17,18,19].

Because of the public’s interest and associated political controversies, much of this work has focused on the application of immunocontraceptives to free-roaming horses. Several studies have yielded data indicating that mares without foals show better body condition than mares with foals, and one has shown increased longevity in PZP-treated mares [13,18]. However, others have suggested that immunocontraception, specifically PZP, could disrupt band stability, increase the harassment of contraceptive-treated mares, and extend or delay foaling seasons [20,21]. There is also evidence that out-of-season births may put foals at risk, potentially disrupting synchrony with resource availability [22]. But some studies have failed to include the presence or absence of foals as a predictor of behavioral effects, or have suggested that immunocontraceptive treatments have consequences separate from their contraceptive effects [17,20,23,24]

This paper uses data from an eight-year study of the effects of PZP application on free-roaming horses in two herd management areas (HMA) managed by the U.S. Bureau of Land Management (BLM) in the western USA to examine the effects of PZP immunocontraception and other factors on the body condition of adult females, the timing of the breeding season, and the survival of offspring of contraceptive-treated and untreated mothers, separating the mare’s treatment status from the presence or absence of foals. This is the first study examining the effects of the PZP-22 delivery protocol, using the single shot, controlled-release preparation of PZP delivered as a primer, on these potential consequences of PZP immunocontraception.

## 2. Materials and Methods

### 2.1. Study Areas

The study was conducted between 2008 and 2015 at Cedar Mountain Herd Management Area (CM), UT, USA and between 2008 and 2014 at Sand Wash Basin Herd Management Area (SWB), CO, USA. The study sites and population parameters are described in detail in [15]. Briefly, CM comprises 1666 km^2^ of sagebrush, grassland, and juniper woodland slopes approximately 80 km west of Salt Lake City, UT, USA. Elevations at CM range from approximately 1400–2460 m. The BLM set the appropriate management level (AML) for CM at 190–390 horses, but the population size from 2009 to 2015 (post-2008 gather) ranged from 397 to 774. SWB comprises 638 km^2^ in northwestern CO, USA, 72 km west of Craig, CO, USA, with elevations ranging from 1900 to 2600 m. AML was set at 163–362 horses, but the actual population size during the study (2009–2014, post-2008 gather) ranged from 227 to 498.

### 2.2. Gathers and PZP Treatments

The CM herd was gathered twice using helicopters during the study, in December 2008 and February 2012. A total of 447 horses was removed from the herd in 2008, and 75 were removed in 2012. PZP-22, consisting of an emulsion of 100 µg PZP dissolved in 0.5 mL PBS and 0.5 mL modified Freunds Complete Adjuvant (mFCA) plus three lactide–glycolide pellets incorporating 450 µg PZP and 900 µg QA-21 (Agenus, Lexington, MA, USA) designed to release at 1, 3, and 12 months, was administered by hand to 70 individually identified (see Section 2.3, below) 2+ year-old mares in the 2008 gather and 85 additional, previously untreated mares in the 2012 gather (PZP for emulsion was provided by the Science and Conservation Center, Billings, MT, USA; PZP-22 pellets were manufactured by J.W. Turner, Jr., at the University of Toledo, Toledo, OH, USA). The boosters of either PZP-22 or ZonaStat-H (both using Freunds Incomplete Adjuvant, FIA, instead of mFCA) were administered by hand in the 2012 CM gather to 58 mares that had originally been treated in 2008. The SWB herd was only gathered once, in October 2008. Following the removal of 280 animals at SWB, 62 mares were hand-injected with PZP-22. Fifty (50) SWB mares treated in 2008 also received dart-delivered boosters of either PZP-22 or ZonaStat-H (both with FIA) in September–November 2010; also included in the analysis of mare body condition, foal birthdates, and mortality were mares treated with ZonaStat-H boosters administered remotely in April–May 2013 (*n* = 23). After gathers, all treated mares were returned to the range at both sites.

Further details of PZP treatment protocols and effectiveness of treatments are provided in [25]. Briefly, however, foaling rates at CM and SWB were reduced by 39–50% in the two years after the initial 2008 treatments, with the poorest effectiveness seen in Year One at SWB. Effectiveness was improved by boosting at both sites, achieving 66–78% for the three years after boosting, with no significant differences in booster effectiveness between ZonaStat-H and PZP-22.

### 2.3. Observations

All observations were carried out on foot or from a vehicle, using binoculars and spotting scopes as needed. Observers averaged 990 field hours per season at CM (2008–2015) and 711 field hours per season at SWB (2008–2014). The length of the field season depended on personnel availability and field conditions. At CM, observations were carried out between March (2011), April (2009–2010) or May (2012–2014) and October (2011–2013) or November (2009, 2010, 2014); at SWB, observations were carried out between April and August (2009), October (2011 and 2012) or November (2010, 2013). Horses were individually identified by group association, location, gender, age, color, face and leg markings, unique identifiers, and mane and tail characteristics. Each positively identified individual received a unique number, with identification and association information incorporated into Excel spread sheets and the Wild Horse Identification Management System (WHIMS; © 2010, Wildwise Solutions). For the horses for which ages were known, ages were determined at gathers from body size and tooth wear, or were known because individuals were born and observed as foals during the study.

Individual foals were matched with their mothers on the basis of nursing and other close affiliative behaviors. Birthdates were established either by direct observation of new foals or by estimates of age by experienced personnel at first sighting. Mortality was confirmed if a carcass was located or euthanasia reported by BLM personnel. Dependent foals not observed with their mothers were presumed dead if they went missing, with the date of disappearance established as the first sighting of the mother without the foal. For other horses, unless there was specific knowledge to the contrary, horses were assumed to be dead or dispersed if they had not been sighted on or adjacent to the HMA at the end of the field season for at least two years.

Body condition was scored visually on a 1–5 scale (recorded in half-units) following [26]. Inter-observer reliability was checked through repeated trials in the field with one of us (KAG) until consistency was achieved. Scores were recorded whenever horses were encountered within approximately 200 m, visual conditions permitting. We attempted to obtain a BCS for each horse at least once a week; scores were used for analysis if at least one score was available for a given month (see Section 2.4).

### 2.4. Analysis

For the purposes of analysis, mares who received only one PZP treatment were defined as “Treated” if the vaccine had been administered within the previous three years (i.e., 2009–2011 for 2008-treated mares at both sites and 2012–2015 for mares treated in February 2012). The boundary was set at three years to reflect the longest-known contraceptive action of a single dose of PZP-22 [27]. Mares receiving either kind of PZP booster in fall 2010 at SWB or spring 2012 at CM were considered treated for the remainder of the study (2012–2015 at CM) and (2011–2014 at SWB), reflecting continued contraceptive effectiveness throughout the period. “Untreated” mares were those that had never been treated with PZP and unboosted mares who had received a PZP-22 treatment more than three years earlier (i.e., 2012 and later for mares initially treated in 2008).

Body Condition Scores (BCS) were initially summarized as monthly averages compiled across the field season for all females ≥ 2 years old for which data were available, pooling across years for each site. We separated treated and untreated mares, and mares with and without foals, and then calculated for each subgroup the change in BCS (ΔBCS) for each female for which sufficient data were available as the average of August–September BCS minus the average of May–June BCS. We then used General Estimating Equations with repeated measures (Generalized Linear Models, IBM SPSS Statistics Version 28) to test for the effects on the ΔBCS of the presence or absence of a foal, PZP treatments, year, and their two-way interaction terms. Model goodness-of-fit was characterized with Corrected Quasi Likelihood under Independence Model Criterion (QICC).

We compared the mortality of the foals of treated and untreated mothers using chi-squared tests, pooling the data from CM and SWB to increase statistical power. For mortality in the first year, we used data from all foaling cohorts (CM: 2008–2015; SWB: 2008–2014). For survival through the second year, we omitted the final cohort year for each site (CM: 2015; SWB: 2014) because we did not have a second year of survival data for those cohorts. To examine the predictors of foal mortality more comprehensively, we conducted logistic regressions with survival as the dichotomous dependent variable and mare treatment status (treated/untreated), foal sex, year, study site, standardized foal birthdate, and maternal age as predictor variables. To create a standardized birth date (SBD) that would allow for the comparisons of foal birthdates across both sites and all years (again to increase power), we calculated the means and standard deviations of foaling dates for each year at each site, then for each foal’s birthdate calculated the number of standard deviations from the mean of that year at that site, i.e., SBD = $\left(X-\bar{x}\right)/s$. SBD’s were then compared between groups using independent *t*-tests, with variances compared with Levene’s test for equality of variances (analysis of raw birthdates gave comparable results.)

All statistical tests were 2-tailed, with α = 0.05 set as the threshold for rejection.

## 3. Results

A total of 316 females (>1 year old) was identified at Cedar Mountains between 2008 and 2015, and 223 females (>1 year old) were identified at Sand Wash Basin between 2008 and 2014. Ages were known for 276 CM females and 211 SWB females.

### 3.1. Mare Body Condition Score Changes

**Figure 1:**
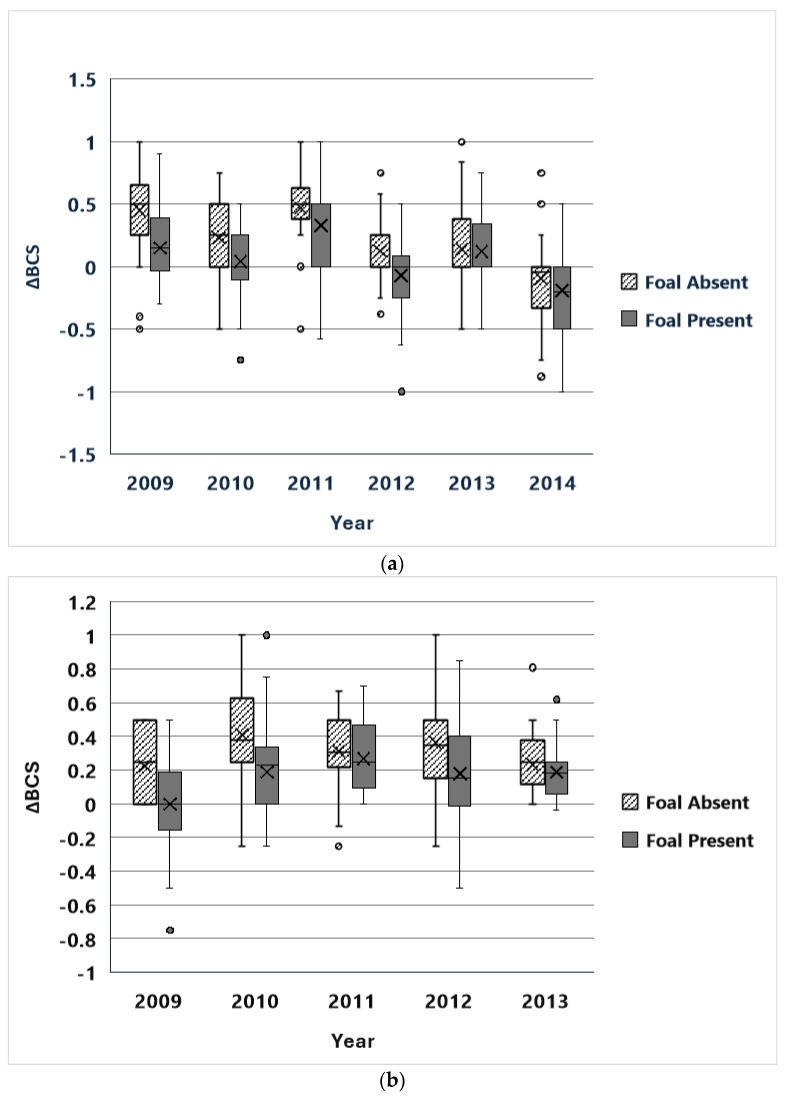
Boxplots of the changes in body condition scores (ΔBCS) across the summer field season in mares with and without foals. BCS values range from 1 to 5 (after [26]), recorded in half-unit increments. (**a**) Cedar Mountain HMA, UT, USA, 2009–2014. (**b**) Sand Wash Basin HMA, CO, USA, 2009–2013.

**Figure 2:**
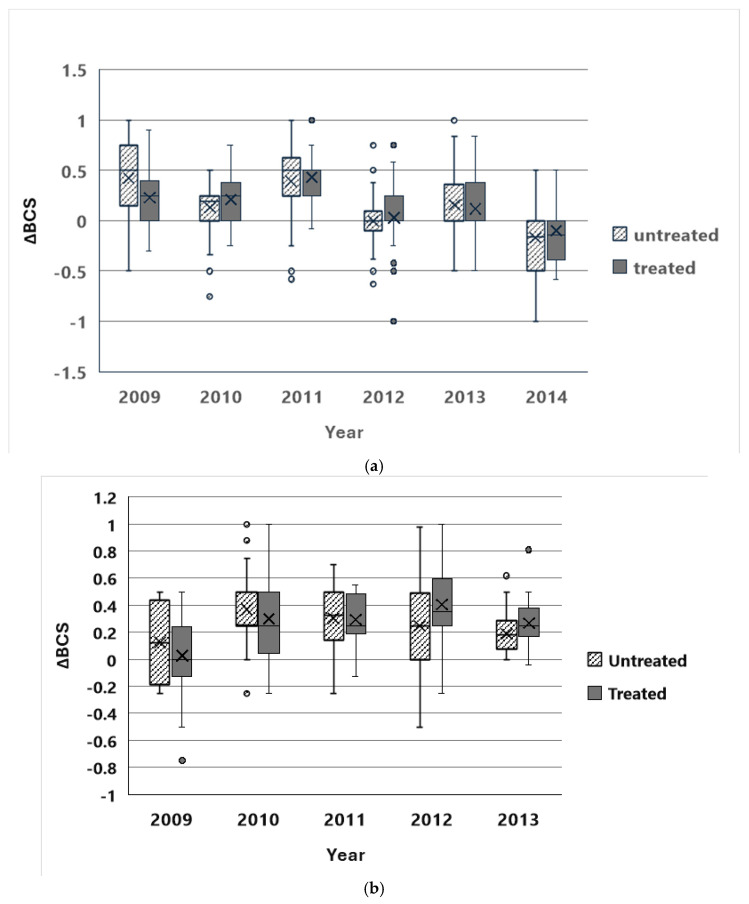
Boxplots of the changes in body condition scores (ΔBCS) across the summer field season in PZP-treated mares and untreated mares. BCS values range from 1 to 5 (after [19]), recorded in half unit increments. (**a**) Cedar Mountain HMA, UT, USA, 2009–2014. (**b**) Sand Wash Basin HMA, CO, USA, 2009–2013.

ΔBCS estimates were available for 234 females at CM and 172 females at SWB. In every year, at both sites, mares without foals improved BCS more than mares with foals over the course of the field season (Figure 1). The direct effects of the PZP-22 treatment protocol on mare body condition were neither consistent nor dramatic (Figure 2).

Multiple-repeated-measure models were run to examine the effects on mare ΔBCS of the presence or absence of foals (“Foal”), PZP treatment within the previous three years (“Treatment”), year of observation (“Year”), and their two-way interaction effects (Table 1). Analyses were run for CM for the years 2009–2014 (*n*= 234 mares) and at SWB for the years 2009–2013 (*n*= 153 mares). At both CM and SWB, the main effects for Foal and Year were highly significant (*p* < 0.001) in every model. No interaction effect was significant in any CM model. At SWB, the Foal by Year and Treatment by Year interaction effects were also significant in some models. The Treatment main effect was not significant in any model at CM or SWB. For CM, the best predictive model judged by QICC included only Year; at SWB the best predictive model included only Foal. For both sites, the second-best model included both Year and Foal main effects.

Thus, the presence or absence of a foal and year-to-year variability were the best predictors of change in mare body condition over the field season. Direct effects of treatment separate from the effects of contraception were not detected.

### 3.2. Effects of PZP Treatments on Foaling Season

**Figure 3:**
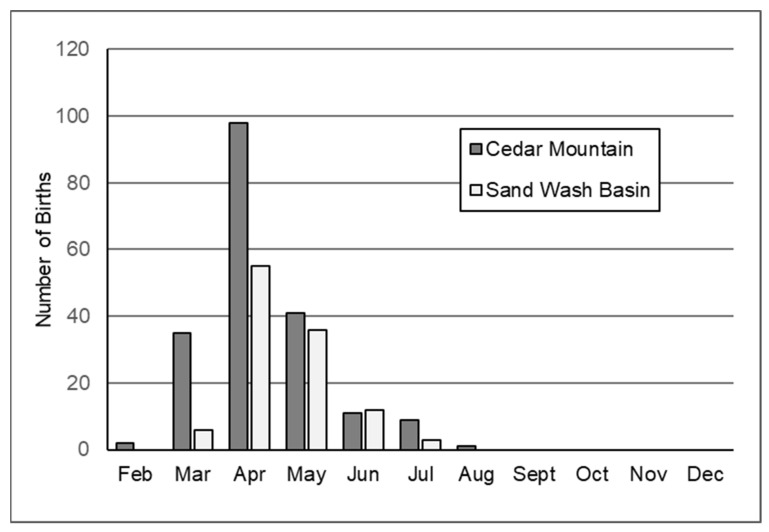
Distribution of births from 2008 to 2009 (pooled) at Cedar Mountains HMA, UT, USA, and Sand Wash Basin HMA, CO, USA. Births during these years were not affected by the initial PZP-22 treatments delivered in December 2008 at CM and October 2008 at SWB.

**Figure 4:**
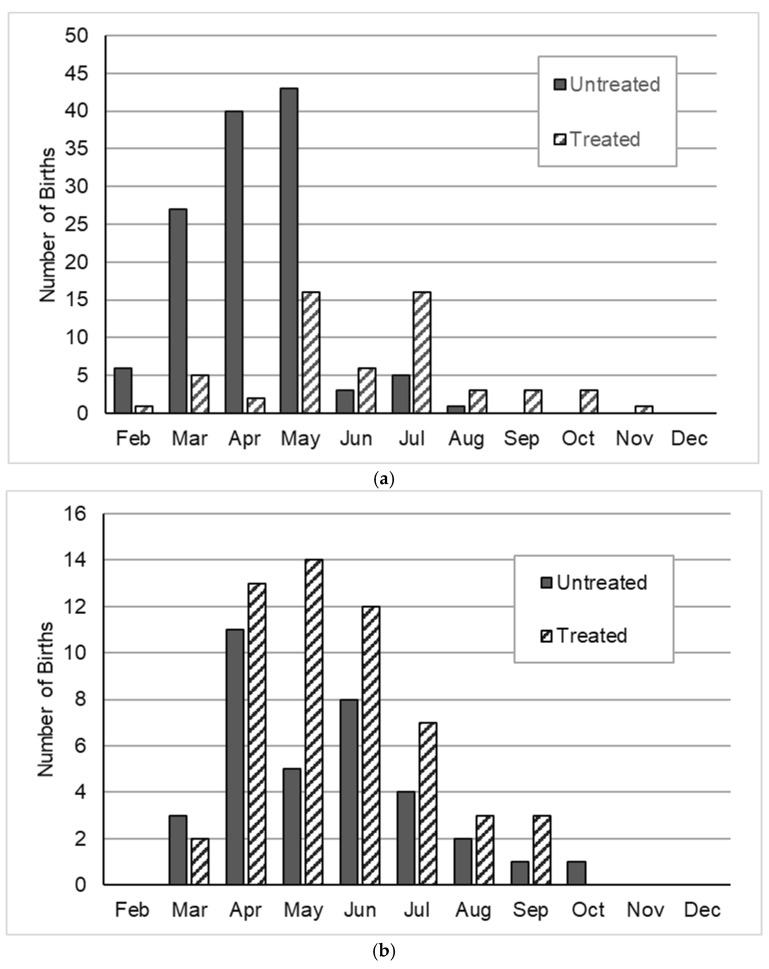
Distribution of births for untreated mares and treated mares following initial PZP-22 injections, 2010–2011 pooled. (**a**) Cedar Mountains HMA, UT, USA; (**b**) Sand Wash Basin, CO, USA.

**Figure 5:**
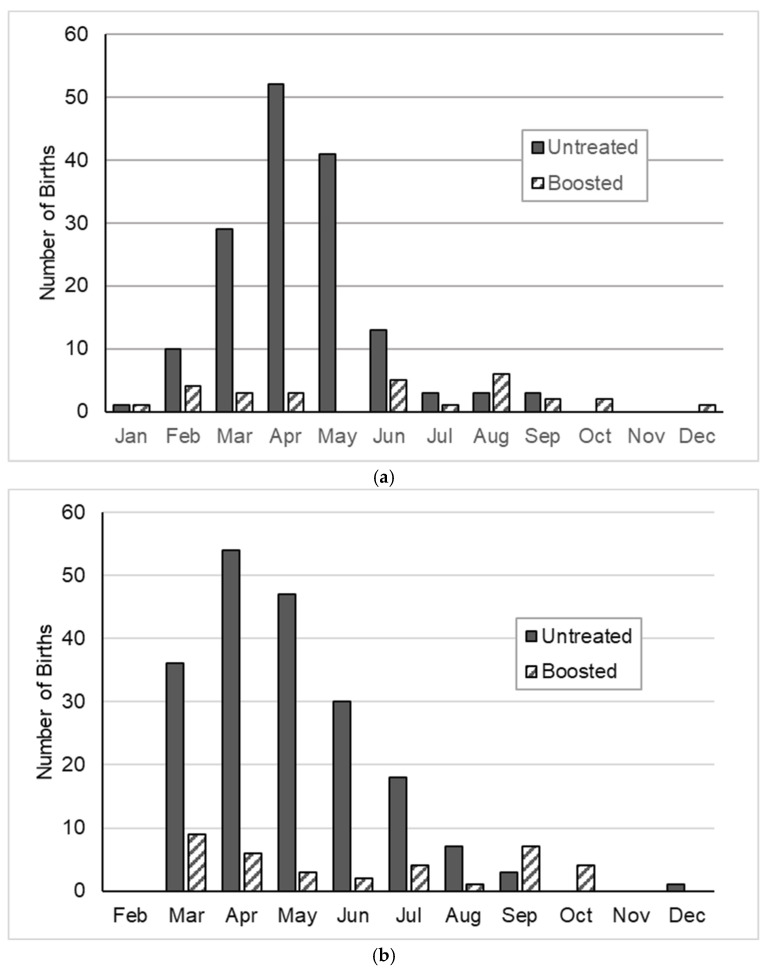
Distribution of births following booster treatments of PZP. (**a**) Cedar Mountains HMA, UT, USA, 2013–2015; boosted February 2012. (**b**) Sand Wash Basin HMA, CO, USA, 2012–2014; boosted autumn 2010.

Birthdates for foals whose mothers were known were recorded at CM from 2008 to 2015 (*n* = 774) and at SWB from 2008 to 2014 (*n* = 433). PZP treatments delayed average birthdates and extended the foaling season at both sites (Figure 3, Figure 4 and Figure 5). In 2008–2009, before the 2008 PZP-22 treatments would have an effect on foaling, 88.3% of births at CM occurred in March through May, and 92.0% of births at SWB occurred in April through June (Figure 3). Subsequent to priming and boosting, births were delayed and spread more widely during the season for both treated and untreated mares.

Pooling results from both sites, standardized birth dates (SBD) of foals of PZP-treated mothers were significantly later in the season than those of the untreated mothers (Treated mothers, SBD = +0.244, Untreated mothers = −0.163; t = 6.93, df = 957, *p* < 0.001). The variance in SBD also differed significantly between the foals of Treated and Untreated mothers (s = 1.09 for PZP-treated females, s = 0.88 for untreated females; Levene’s test for equality of variances, F = 35.166, df = 1, 1202, *p* < 0.001).

However, breaking down the analysis by site and primer vs. booster, the SBD’s of foals of treated mares were significantly later and more widely distributed than those of foals of untreated mares at CM for both primers (Figure 4a) and boosters (Figure 5a) (2010–2011, t = −5.57, df = 124.2, *p* < 0.001; Levene’s test of equal variances, F = 15.35, df = 1224, *p* < 0.001; 2013–2015, t = 6.00, df = 181, *p* < 0.001; Levene’s test of equal variances, F = 67.42, df = 1278, *p* < 0.001). At SWB, however, differences in the distribution of SBD’s between treated and untreated mares were more inconsistent. Birthdates in 2010–2011 were significantly later in treated than untreated mares, but the difference in SBD spread was not quite significant (Figure 4b; t = −3.24, df = 197, *p* = 0.001; Levene’s test of equal variances, F = 3.41, df = 1, 197, *p* = 0.066). For the SWB boosters (2012–2014), neither means nor variances differed between treated and non-treated mares (Figure 5b; t = −1.08, df = 230; Levene’s test of equal variances, F = 2.06, df = 1, 230, *p* = 0.283).

Of 775 foals whose mothers were known at CM from 2008 to 2015, 24 (3.1%) died in their first year, with an additional 7 known to have died as yearlings. At SWB, a total of 21 of 433 foals (4.8%) whose mothers were known died in their first year in 2008–2014, with 3 additional known to have died as yearlings.

Mortality rates for the first and second year were virtually identical for the foals of PZP-treated and untreated mothers. At CM, first year mortality for foals of PZP-treated females was 3.5%; for untreated females it was 2.8% (*n* = 775; χ^2^ = 0.351, df = 1, *p* = 0.533). For SWB, first year mortality for foals of PZP-treated females was 4.4%; for untreated females it was 5.5% (*n* = 433, χ^2^ = 0.160, df = 1, *p* = 0.689). At CM, mortality through the second year for foals of PZP-treated females was 4.0%; for untreated females it was 4.1% (*n* = 640; χ^2^ = 0.002, df = 1, *p* = 0.962). For SWB, mortality through the second year for foals of PZP-treated females was 6.1%; for untreated females it was 5.9% (*n* = 334; χ^2^ = 0.005, df = 1, *p* = 0.941).

As noted above, the birthdates of foals of PZP-treated mothers were both later on average and more spread out over the year than those of untreated mothers, although less clearly at SWB. Consistent with the findings indicating no maternal treatment effects on foal mortality, however, the mean birthdates of foals dying vs. surviving their first year and dying vs. surviving their second year did not differ (first year, t = 0.912, df = 1207, *p* = 0.362; second year, t = 0.384, df = 971, *p* = 0.701).

To search more widely for potential causes of first- and second-year mortality and to confirm the results of the simple analysis above, we conducted logistic regressions on the pooled mortality data from both sites using foal sex, SBD, year, study site, and maternal PZP treatment status and age as independent variables (survival through first year, *n* = 910; survival through second year, *n* = 702). None of these variables significantly predicted survival through the first year (Table 2a). However, survival through the second year was significantly predicted by maternal age; (*p* = 0.016) (Table 2b).

Further examining the impact of maternal age on foal survival, we determined that maternal age for foals that survived their first year was significantly greater than the maternal age for foals dying their first year (6.36 ± 4.00 y vs. 4.23 ± 2.05 y; *t*-test with unequal variances, t = 2.75, df = 48.4, *p* = 0.008). Consistent with the logistic regression results, mean maternal age for foals surviving through their second year was significantly greater than the mean maternal age for foals dying before the end of their second year (6.37 ± 4.01 y vs. 4.43 ± 2.10 y; *t*-test with unequal variances, t = 5.40, df = 61.79, *p* < 0.001).

Thus, survival through both the first and second years was significantly greater for foals of older mares, but neither maternal PZP-treatment status, foal birthdate, or any other variable examined predicted foal mortality.

## 4. Discussion

In this study, conducted over eight years on more than 500 wild horse mares and their offspring at two different HMA’s in the western USA, we did not find evidence that initial treatment with PZP-22 or subsequent boosting with PZP-22 or ZonaStat-H reduced the body condition of treated mares or increased foal mortality over the first two years. The body condition of mares without foals increased faster than the body condition of mares with foals across the field season, from late spring to fall. When statistically separated from its contraceptive effects, the PZP-22 primer PZP-22/ZonaStat-H treatment protocol followed here did not otherwise influence female body condition. Overall, the foal mortality was low in these two PZP-treated herds, with only 45 foal deaths reported in 1207 births (3.7%) at the two sites, and mortality rates of foals of treated and non-treated females were essentially identical. Birthdate did not affect risk of foal mortality through the first two years of life.

Although year-to-year variation was a highly significant predictor of changes in body condition scores, the more rapid improvement of body condition observed in mares without foals was observed every year at both sites. It was somewhat less pronounced at SWB, possibly because BCS’s were generally higher there. Horses at SWB had more water sources available to them than CM horses, and thus had to travel shorter distances to reach water and were able to forage more freely across the landscape than CM horses. Improvements in mare body condition were also reported among mares in a wild horse herd treated with ZonaStat-H at Assateague Island National Seashore, Maryland, also with better body condition scores for non-lactating than lactating females [13]. Ransom et al. [18] also found that mares with foals showed lower body condition than mares without foals in three small Western USA herds treated with ZonaStat-H.

Initial PZP-22 treatments and subsequent boosters were associated with a delay and extension of the foaling season. The lengthening of the foaling season, and by implication, the mating season, has also been previously documented in wild horse herds treated with ZonaStat-H. Nunez et al. [21], reporting an analysis of 162 births from 1995–1997 and 2000–2008 on Shackleford Island, NC, found that foaling occurred over a wider range of dates after contraception treatments with ZonaStat-H began in 2000 and that, similar to what was observed in the current study, the range of birthdates was wider and later for PZP-treated mares than for untreated mares. Ransom et al. [22] also found the lengthening of the foaling season associated with PZP treatments. In contrast, Kirkpatrick and Turner [28] failed to find a statistically significant lengthening of the birth season for 168 foals born at Assateague Island between 1986 and 2002, although birth seasons were trending longer for foals of treated mares.

The lengthening and delay of the foaling season in PZP-treated animals was more pronounced at CM, and less so at SWB. In part, this may be related to consistently greater vaccine effectiveness at CM than at SWB, and partially because the initial 2008 PZP-22 vaccinations at SWB were ill-timed (PZP-22 is optimized for delivery from late December to March, and the 2008 SWB treatments were delivered in October), initial treatments were relatively ineffective [25]. Booster effectiveness was also higher at CM than at SWB; still, it is a little surprising that we found no significant differences at SWB in foaling season length or duration between treated and untreated mares (Figure 4b and Figure 5b).

Despite concerns raised about the risks posed to foals by late births [21,22] (which were more common for treated animals but also present in untreated animals), the risks of mortality through the first and second years of life were essentially identical for foals of mares treated with PZP-22 or a booster in the previous three years and foals of never-treated mares and mares that hadn’t been treated for more than three years. We also failed to detect at these sites any evidence that foals born late in the season were at greater risk of dying through their first two years (Table 2). These results parallel those found for mares treated with ZonaStat-H at Assateague Island [28]. Ransom et al. [22], studying PZP-treated mares in three smaller Western US herds, found that there was a greater risk of mortality for foals born later in the year, post-green foliage peak, and additionally linked mortality risk to mare condition. Foal mortality was uniformly higher in these herds than in the current study—15% vs. less than 4%—and mares appeared to display a wider range of body conditions than in the current study, though the BCS scale used was different. This contrast suggests that the effects of contraception on timing of breeding are likely to act differently on foal mortality depending on resource availability and mare body condition. Nunez et al. [21], anecdotally reporting improved body condition in PZP-treated mares, note that this improved body condition could provide PZP-treated mares with more flexibility in their energy budget for timing reproduction. Our observations regarding vaccine effectiveness and the distribution of births are consistent with that hypothesis, and high survival rates among all foals at CM and SWB likewise imply that whatever behavioral or nutritional costs might be associated with late births could be offset by improved body condition of contracepted mares if resource conditions permit.

Lower survival rates in foals of younger females are well-documented in wild horses [29,30], and our findings that mortality in the first two years of life decrease with maternal age are consistent with the previous literature. The U.S. National Park Service’s management plan for application of ZonaStat-H to regulate the wild horse population at Assateague Island National Seashore provides for initial ZonaStat-H treatments of young mares and then release from contraception, which could provide a survival advantage to foals [28].

## 5. Conclusions

One should be cautious about extrapolating to wild horses living in other environments the findings reported here on the positive effects of the PZP-22 contraception protocol on mare body condition and the absence of effects on foal survival. In the Cedar Mountain and Sand Wash Basin study areas, overall foal and yearling mortality were low, and in environments such as those described in [22], which may have more limited forage availability or higher population densities, foals born outside normal foaling windows might be subject to higher mortality risks. Nevertheless, the reduced energetic burden on mares treated with effective, reversible, non-invasive contraceptives such as PZP might help buffer individual female wild horses and their herds against environmental extremes, including those anticipated as global climate change worsens.

## Figures and Tables

**Table 1:** Changes in female Body Condition Score (ΔBCS) as a function of the presence or absence of foal (“Foal”), PZP treatment status (“treated within the last three years”) and year of observation (“Year”). QICC = corrected quasi likelihood under the independence model criterion. (a) Cedar Mountains HMA, UT, USA (2009–2014; *n* = 234); (b) Sand Wash Basin HMA, CO, USA (2009–2013; *n* = 153).

**(a)**							
**Model**	**Foal**	**Year**	**Treated Within 3 Years**	**Year by Foal**	**Year by Treated**	**Foal by Treated**	**QICC**
All Main effects + two-way interactions	χ^2^ = 27.52 df = 1 *p* < 0.001	χ^2^ = 143.60 df = 5 *p* < 0.001	χ^2^ = 0.67 df = 1 *p* = 0.413	χ^2^ = 6.71 df = 5 *p* = 0.243	χ^2^ = 5.64 df = 5 *p* = 0.343	χ^2^ = 0.006 df = 1 *p* = 0.940	96.512
Foal and Year Main effects	χ^2^ = 35.47 df = 1 *p* < 0.001	χ^2^ = 149.13 df = 5 *p* < 0.001					74.223
Foal only	χ^2^ = 17.94 df = 1 *p* < 0.001						80.134
Year only		χ^2^ = 102.16 df = 5 *p* < 0.001					62.556
**(b)**							
**Model**	**Foal**	**Year**	**Treated Within 3 Years**	**Year by Foal**	**Year by Treated**	**Foal by Treated**	**QICC**
All Main effects + two-way interactions	χ^2^ = 45.52 df = 1 *p* < 0.001	χ^2^ = 20.55 df = 4 *p* < 0.001	χ^2^ = 1.56 df = 1 *p* = 0.212	χ^2^ = 25.73 df = 4 *p* < 0.001	χ^2^ = 13.33 df = 4 *p* = 0.010	χ^2^ = 0.00 df = 1 *p* = 0.971	56.740
Foal and Year Main effects only	χ^2^ = 43.77 df = 1 *p* < 0.001	χ^2^ = 33.09 df = 4 *p* < 0.001					38.655
Foal only	χ^2^ = 56.91 df = 1 *p* < 0.001						32.527
Year only		χ^2^ = 47.61 df = 4 *p* < 0.001					39.139

**Table 2:** Logistic regressions of foal survival at Sand Wash Basin HMA, CO, USA and Cedar Mountains HMA, UT, USA (study sites pooled). (a) Survival through year one (SWB, 2008–2014; CM, 2008–2015; *n* = 910), neither maternal age nor treatment status, year, foal sex, standardized birthdate, or study site significantly predicted foal survival. (b) Survival through year two (SWB, 2008–2013; CM, 2008–2014; *n* = 698). The mother’s age significantly predicted survival, while the PZP treatment status, year, foal sex, standardized birth date, and study site did not.

**(a)**						
**Variable**	**B**	**SE**	**Wald χ^2^**	**df**	***p***	**Exp(B)**
Standardized Birth Date	0.059	0.171	0.120	1	0.729	1.061
Maternal Age	−0.084	0.059	2.042	1	0.153	0.919
Year	−0.005	0.082	0.003	1	0.955	0.995
Foal Sex	0.482	0.350	1.902	1	0.168	1.620
Maternal Treatment Status	−0.165	0.357	0.213	1	0.644	0.848
Study Site	0.266	0.343	0.603	1	0.438	1.305
Constant	6.427	165.948	0.001	1	0.969	618.271
**(b)**						
**Variable**	**B**	**SE**	**Wald χ^2^**	**df**	***p***	**Exp(B)**
Standardized Birth Date	−0.020	0.177	0.013	1	0.910	0.980
Maternal Age	−0.168	0.070	5.773	1	0.016	0.845
Year	0.116	0.101	1.310	1	0.252	1.123
Foal Sex	0.239	0.345	0.480	1	0.488	1.270
Maternal Treatment Status	0.027	0.374	0.005	1	0.943	1.027
Study Site	0.436	0.349	1.564	1	0.211	1.547
Constant	−235.714	203.996	1.335	1	0.248	0.000

## Data Availability

Dataset available on request from the authors.
